# ER stress causes widespread protein aggregation and prion formation

**DOI:** 10.1083/jcb.201612165

**Published:** 2017-08-07

**Authors:** Norfadilah Hamdan, Paraskevi Kritsiligkou, Chris M. Grant

**Affiliations:** Faculty of Biology, Medicine and Health, The University of Manchester, Manchester, England, UK

## Abstract

Disturbances in endoplasmic reticulum (ER) homeostasis create a condition termed ER stress. This activates the unfolded protein response (UPR), which alters the expression of many genes involved in ER quality control. We show here that ER stress causes the aggregation of proteins, most of which are not ER or secretory pathway proteins. Proteomic analysis of the aggregated proteins revealed enrichment for intrinsically aggregation-prone proteins rather than proteins which are affected in a stress-specific manner. Aggregation does not arise because of overwhelming proteasome-mediated degradation but because of a general disruption of cellular protein homeostasis. We further show that overexpression of certain chaperones abrogates protein aggregation and protects a UPR mutant against ER stress conditions. The onset of ER stress is known to correlate with various disease processes, and our data indicate that widespread amorphous and amyloid protein aggregation is an unanticipated outcome of such stress.

## Abstract (precis)

ER stress results in widespread aggregation of proteins that are not localized to the ER or are part of the secretory system. Hamdan et al. demonstrate that amorphous and amyloidogenic protein aggregation is an indirect consequence of perturbing ER homeostasis.

## Introduction

The ability to maintain the balance between protein biogenesis, folding, trafficking, and degradation in the face of changing conditions is essential for viability in all eukaryotic cells. This is illustrated by the plethora of human diseases influenced by alterations in protein homeostasis (Labbadia and Morimoto, 2015). The ER is the first organelle required for folding and trafficking of nascent membrane proteins and proteins that enter the secretory pathway. Proteins enter in an unfolded state, and ER-resident enzymes facilitate their oxidative folding, modification, and trafficking reactions (Walter and Ron, 2011). An ER stress occurs when there is a breakdown in ER protein homeostasis.

ER-associated degradation (ERAD) acts to remove proteins that do not fold properly from the ER. Proteins are retrotranslocated into the cytosol, where they are ubiquitinated and degraded by the proteasome (Nakatsukasa and Brodsky, 2008; Christianson and Ye, 2014; Zattas and Hochstrasser, 2015). Loss of ERAD results in an accumulation of misfolded ER proteins and promotes ER stress (Walter and Ron, 2011). Substrate recognition and membrane extraction is coordinated by ER-embedded ubiquitin ligase complexes; three distinct membrane protein complexes define different ERAD pathways (L-lumen, M-membrane, and C-cytosol), depending on the localization of the degradation signal (Carvalho et al., 2006; Nakatsukasa and Brodsky, 2008; Ruggiano et al., 2014). In yeast, ERAD-L and ERAD-M substrates are targeted for degradation by the Hrd1 complex, whereas, ERAD-C substrates are recognized by the Doa10 complex (Brodsky and Skach, 2011; Thibault and Ng, 2012; Christianson and Ye, 2014; Ruggiano et al., 2014; Zattas and Hochstrasser, 2015). The unfolded protein response (UPR) is a signaling pathway that is activated in response to ER stress by an ER-localized kinase, Ire1 (Travers et al., 2000). Ire1 senses the accumulation of unfolded proteins in the ER and acts as a specific endoribonuclease, splicing the *HAC1* mRNA (Sidrauski and Walter, 1997). The translation product of spliced *HAC1* mRNA is the transcriptional activator for genes affecting protein folding, degradation, and trafficking to restore ER homeostasis (Travers et al., 2000; Walter and Ron, 2011).

Although much is now known regarding the role of ER stress defense systems in maintaining ER protein homeostasis, little is known regarding the consequences of ER dysfunction on protein homeostasis in other cellular compartments. In the current study, we show that ER stress results in widespread cytoplasmic protein aggregation, including both amorphous and amyloidogenic aggregation.

## Results and discussion

### ER stress causes widespread protein aggregation

**Figure 1.**
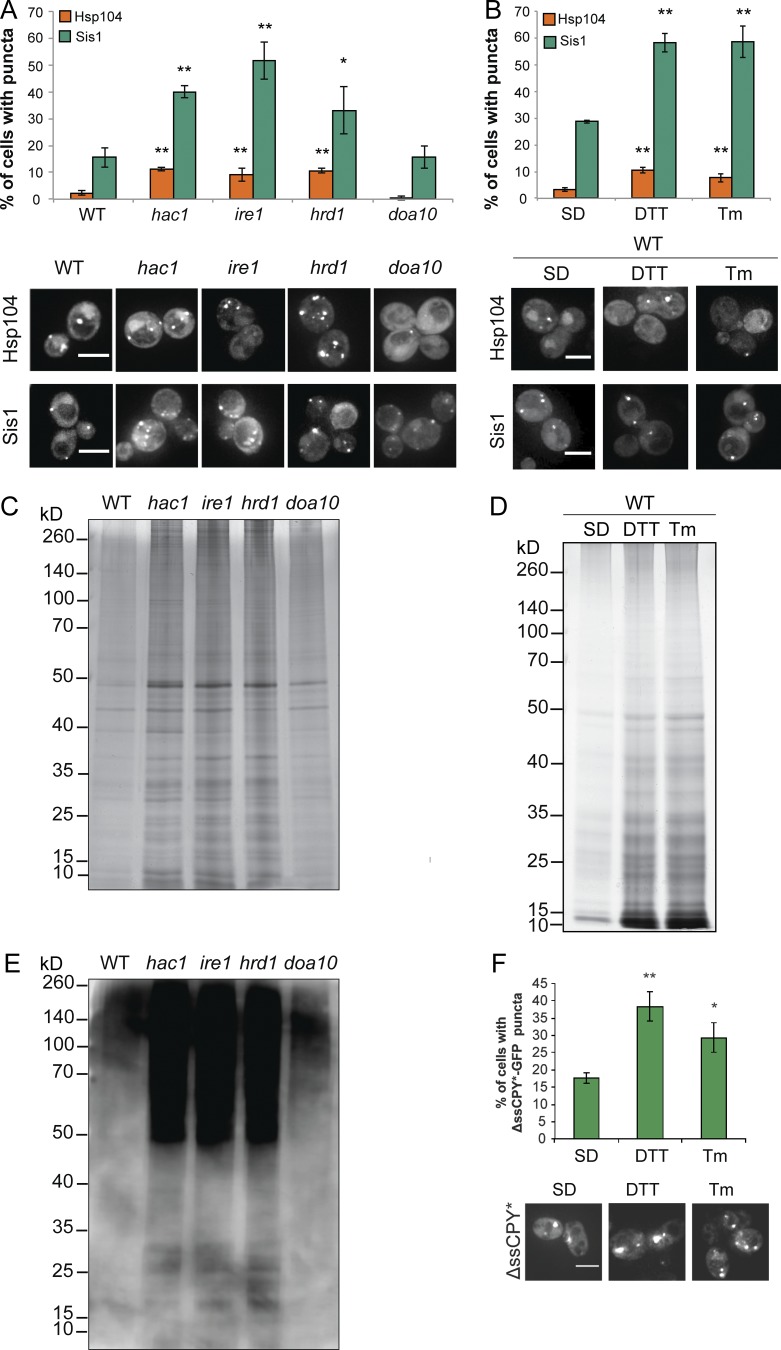
**ER stress causes protein aggregation.** (A) Hsp104-RFP and Sis1-GFP were visualized in wild-type, *hac1, ire1, hrd1*, and *doa10* mutant cells and in wild-type cells exposed to DTT or Tm. Bars, 4 µm. (B) The percentage of cells containing puncta is quantified for each strain from three independent biological repeat experiments ± SD. (C and D) Silver staining of protein aggregates isolated from the same strains as in A and B. (E) Protein ubiquitination was analyzed in the isolated protein aggregates by Western blot using an α-ubiquitin antibody. (F) Examples of cells containing ΔssCPY^∗^-GFP puncta in a wild-type strain after DTT or Tm treatment. *, P < 0.05; **, P < 0.01; ***, P < 0.005 (*n* = 3).

Protein aggregation was analyzed in mutants deficient in the UPR (*HAC1* and *IRE1*) or ERAD (*HRD1* and *DOA10*) and in cells exposed to tunicamycin (Tm) or DTT to promote ER stress (Cox et al., 1993; Kohno et al., 1993). Foci of protein aggregates can be detected using the fluorescently tagged Hsp104 disaggregase or Sis1 Hsp40 chaperone (Lum et al., 2004; Erjavec et al., 2007; Lee et al., 2010; Malinovska et al., 2012; Park et al., 2013). The number of cells containing Hsp104-RFP or Sis-GFP puncta was significantly increased in *hac1*, *ire1*, and *hrd1* mutants (Fig. 1 A) and in wild-type cells exposed to DTT or Tm (Fig. 1 B). To confirm that protein aggregation occurs during ER stress, aggregates were purified using an established biochemical approach (Tomoyasu et al., 2001; Jang et al., 2004; Rand and Grant, 2006; Koplin et al., 2010). Elevated protein aggregation was observed in *hac1*, *ire1*, and *hrd1* mutant strains (Fig. 1 C) and in wild-type cells exposed to DTT or Tm (Fig. 1 D). Ubiquitinated proteins were increased in the aggregate fractions isolated from the *hac1*, *ire1*, and *hrd1* mutants, suggesting that aggregated proteins are targeted to the proteasome for degradation (Fig. 1 E). To further confirm that protein aggregates form during ER stress, a mutant version of the secretory protein carboxypeptidase Y lacking its signal sequence (ΔssCPY^∗^) was used, which is rapidly degraded by the ubiquitin proteasome system (UPS; Eisele and Wolf, 2008; Park et al., 2013). ΔssCPY^∗^-GFP aggregate formation was elevated in response to ER stress imposed by DTT or Tm treatment (Fig. 1 F).

### ER stress causes the aggregation of aggregation-prone proteins rather than specific ER targets

**Figure 2.**
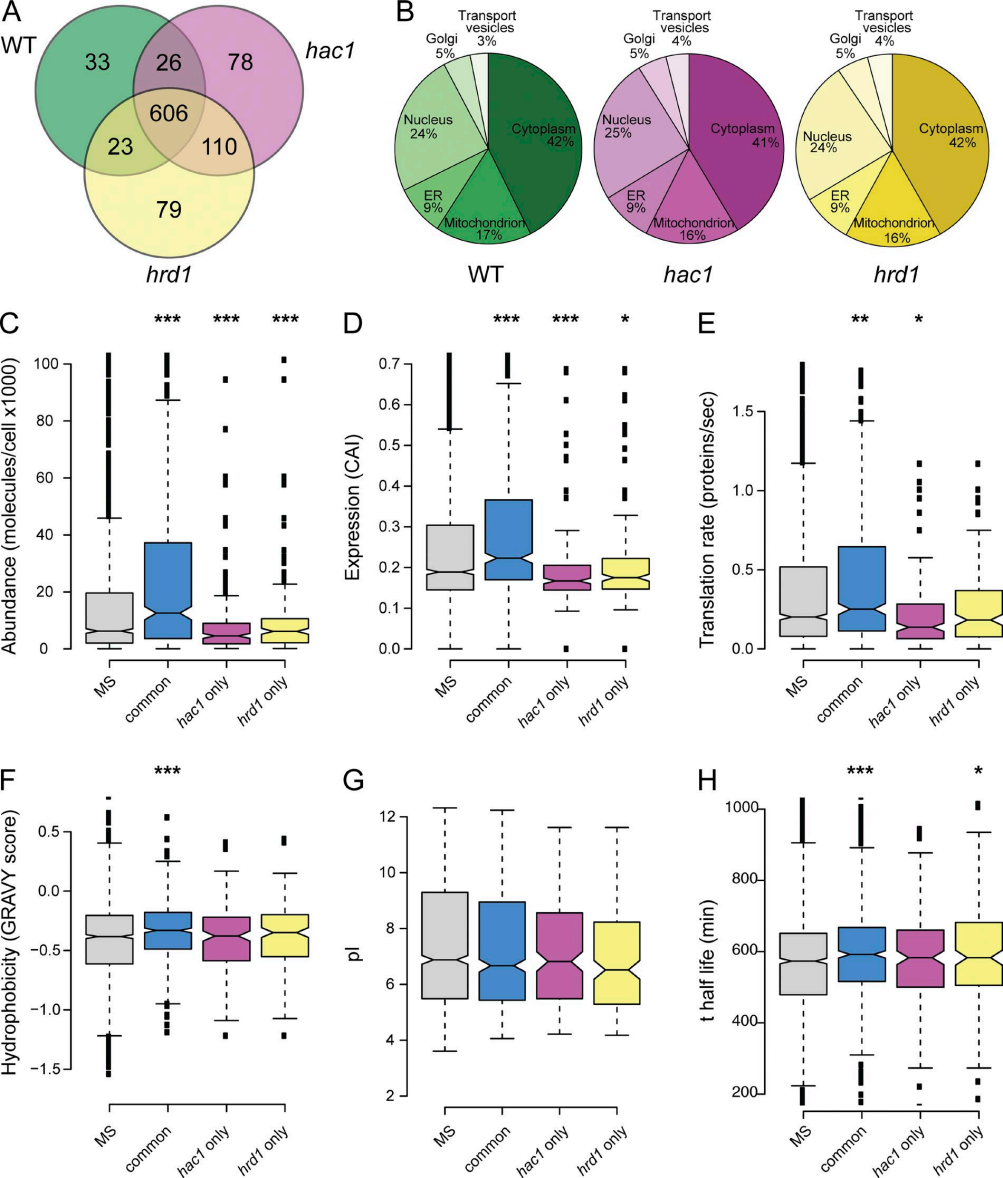
**Aggregated proteins in wild-type and ER stress mutants have similar localization and physicochemical properties.** (A) Venn diagram showing the overlaps between proteins aggregating in wild-type (green), *hac1* (pink), and *hrd1* (yellow) mutant strains. (B) Diagrams showing the localization of the proteins aggregating in wild-type, *hac1*, and *hrd1* strains. (C–H) Box plots showing comparisons of physicochemical properties for the aggregated proteins common to the wild-type, *hac1*, and *hrd1* mutant strains (common), present in the *hac1* mutant, but not the wild-type, strain (*hac1* only), and present in the *hrd1* mutant, but not the wild-type, strain (*hrd1* only). Aggregated proteins were compared with unaggregated proteins (MS). (C) Protein abundance. (D) Codon adaptation index (CAI). (E) Translation rates. (F) Grand mean of hydrophobicity (GRAVY). (G) Isoelectric points (pI). (H) Protein stability. Mann–Whitney *U* tests were used to assess the statistical significance of observed differences; *, P < 0.05; **, P < 0.01; ***, P < 0.005.

Aggregated proteins were identified using mass spectrometry. A large overlap in the proteins that aggregate in wild-type, *hac1*, and *hrd1* mutants strains was observed, indicating that the majority of proteins do not aggregate in a mutant-specific manner (Fig. 2 A). Similar functional categories were enriched within aggregate fractions prepared from wild-type and ER stress mutants (Fig. S1), including common enrichment in major cellular processes such as metabolism, energy, protein synthesis, and protein fate. Strikingly, no enrichment for ER-related processes was observed in *hac1* or *hrd1* mutant strains. The majority of aggregated proteins were predicted to localize to the cytoplasm, and no enrichment for ER or secreted proteins was observed in *hac1* or *hrd1* mutant strains compared with the wild-type strain (Fig. 2 B).

We assessed the physicochemical properties of aggregated proteins to determine whether they possess particular properties that make them aggregation prone. The 606 proteins that commonly aggregated in the wild-type, *hac1*, and *hrd1* mutant strains (common set) were compared with the 189 and 190 proteins that aggregated in the *hac1* and *hrd1* mutants, but not in the wild-type strain. No functional classes were enriched in the *hac1* or *hrd1* mutants that were not also enriched in the common set (Fig. S2). The aggregated proteins were compared with a list of yeast proteins detectable by mass spectrometry (MS; MS set) to represent the properties of unaggregated proteins (Washburn et al., 2001). The common set was enriched for proteins with higher abundance (molecules per cell), higher expression levels (codon adaption index), and higher translation rate compared with the MS set (Fig. 2, C–E). In contrast, the proteins that specifically aggregated in *hac1* or *hrd1* mutants tended to be have lower abundances and translation rates compared with the MS set (Fig. 2, C–E). Hydrophobicity indicates a propensity to aggregate (Vabulas et al., 2010; Tamás et al., 2014), and we found that proteins in the common set showed a significant increase in hydrophobicity (GRAVY score) compared with the MS set, whereas the proteins which aggregate in *hac1* or *hrd1* mutants were similar to the MS set (Fig. 2 F). We found no differences in isoelectric points (pI) between the aggregated proteins and the MS set (Fig. 2 G). Using a global proteome turnover database (Christiano et al., 2014), we found that proteins in the common set and *hrd1-*only sets show, on average, a longer half-life than proteins in the MS proteome, suggesting that these proteins are normally stable in their native folded states (Fig. 2 H). Collectively, these data indicate that the proteins that commonly aggregate in the wild-type, *hac1*, and *hrd1* mutants have common properties indicative of aggregation-prone proteins.

### Protein aggregation does not arise because of inhibition of UPS-mediated protein degradation

**Figure 3.**
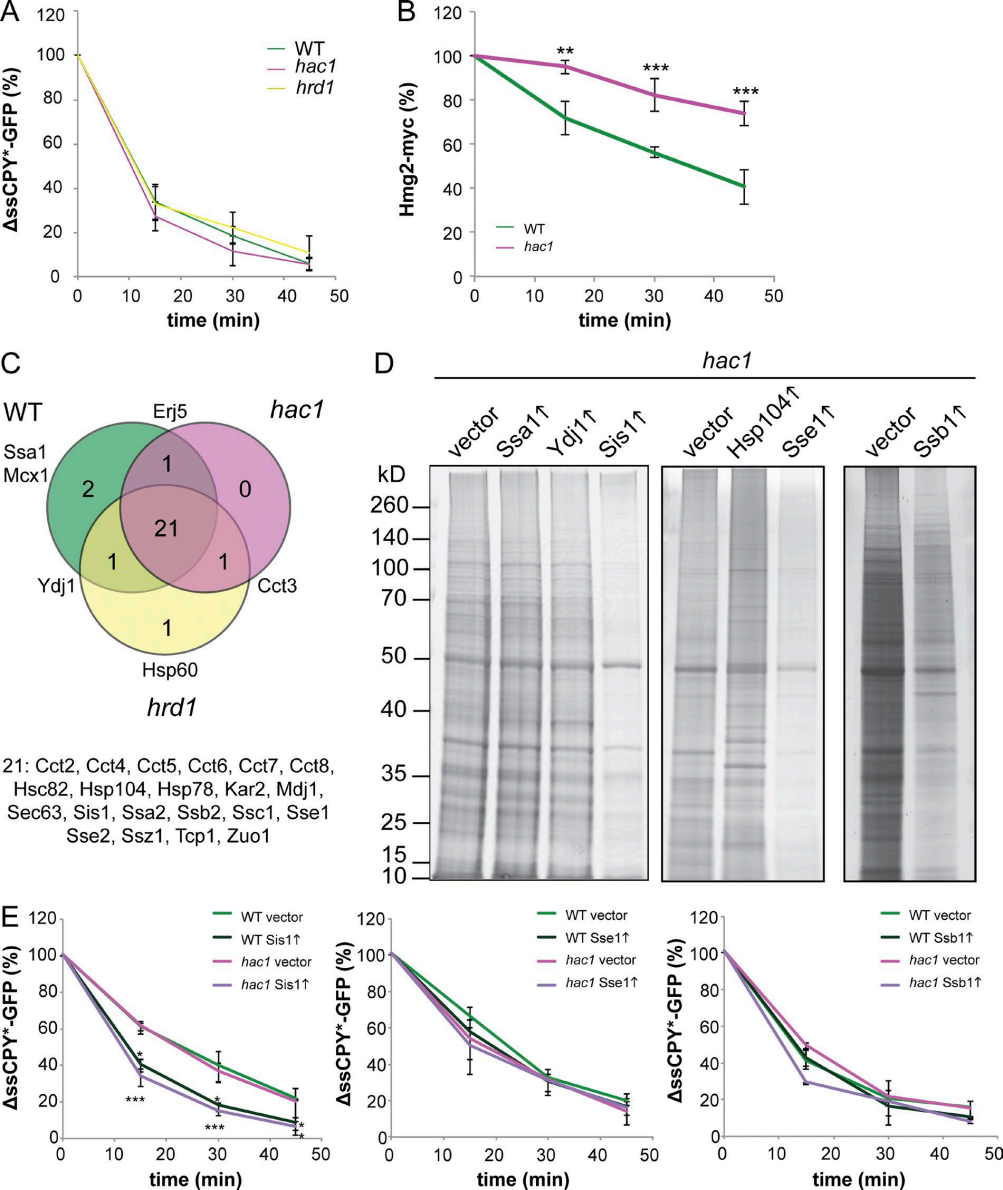
**Selected chaperones reduce protein aggregation in a *hac1* mutant.** (A) Turnover rate of ΔssCPY^∗^-GFP in wild-type, *hac1*, and *hrd1* strains (*n* = 3) ± SD. (B) Turnover rate of Hmg2-myc in wild-type and *hac1* strains (*n* = 3) ± SD. (C) The overlap between chaperones identified within the protein aggregate datasets of the wild-type, *hac1*, and *hrd1* strains. The 21 chaperones listed were present in the aggregates of all strains. (D) Silver staining of protein aggregates isolated from the *hac1* mutant containing galactose-regulatable expression plasmids for Ssa1, Ydj1, Sis1, Hsp104, Sse1, or Ssb1. Vector denotes an empty vector control. (E) Turnover rate of ΔssCPY^∗^-GFP in wild-type and *hac1* strains containing galactose-regulatable expression plasmids for Sis1, Sse1, or Ssb1 or empty vector controls (*n* = 3) ± SD. *, P < 0.05; **, P < 0.01; ***, P < 0.005 (*n* = 3).

We considered two mechanisms that might account for the high levels of protein aggregation in ER stress mutants. First, protein aggregation might arise because of a defect in proteasomal degradation that could result in the accumulation of misfolded proteins and subsequent aggregation rather than turnover. For example, an inhibition of UPS-mediated protein degradation might occur if ER stress generates more substrates than the UPS can cope with, effectively overwhelming the degradation machinery. We tested this possibility by examining the degradation kinetics of the ΔssCPY^∗^-GFP reporter as a model substrate for UPS-mediated degradation (Park et al., 2007, 2013). Rapid turnover of ΔssCPY^∗^-GFP was observed in the wild-type strain as well as in the *hac1* or *hrd1* mutants, suggesting that there is no defect in UPS degradation (Fig. 3 A). For comparison, we examined the turnover of Hmg2 (HMG-CoA reductase), a well-characterized ERAD substrate (Hampton et al., 1996), and we found that its turnover is slower in a *hac1* mutant than in a wild-type strain (Fig. 3 B).

### Overexpression of selected chaperones prevents ER stress–induced protein aggregation

The second possibility that we considered was that proteins that are protected from misfolding by the cellular protein quality control systems might accumulate and aggregate if these are disrupted or become overwhelmed. To counteract protein aggregation, cells contain many molecular chaperones (Flynn et al., 1991; Hartl et al., 2011; Verghese et al., 2012), but increased aggregation can arise because of insufficient availability of chaperones when ERAD substrates and aggregates accumulate. Increased cytosolic aggregation might therefore arise in ER stress mutants if protein aggregates or ERAD substrates sequester key chaperones, limiting their availability to maintain protein homeostasis. Of the 63 known chaperones in *Saccharomyces cerevisiae* (Gong et al., 2009), we identified 27 distributed between all the datasets (Fig. 3 C). These chaperones presumably localize to aggregates to mitigate any toxic consequences, so we examined whether the aggregates are enriched in proteins that have increased chaperone interactions. We found no enrichment for proteins with extensive chaperone interactions in the aggregated proteins compared with unaggregated proteins (Fig. S3 A).

We next tested whether overexpression of candidate chaperones identified in our aggregate fractions could prevent aggregate formation. Overexpression of Hsp104, Ssa1, or Ydj1 did not affect protein aggregation, whereas Sis1, Sse1, and Ssb1 dramatically reduced the levels of aggregation in a *hac1* mutant (Fig. 3 D). Western blot analyses confirmed that chaperones were overexpressed (Fig. S3 B). The ΔssCPY^∗^-GFP reporter undergoes rapid UPS-mediated degradation dependent on cytosolic Hsp70 (Ssa1) and Hsp40’s (Ydj1 and Sis1; Park et al., 2007, 2013); thus, we tested whether chaperone overexpression affects UPS-mediated degradation (Fig. 3 E). Overexpression of Sis1 increased the rate of degradation of ΔssCPY^∗^-GFP in both the wild-type and *hac1* mutant strains, in agreement with the idea that Sis1 is required for targeting misfolded proteins for degradation by the UPS system (Park et al., 2013; Shiber et al., 2013; Summers et al., 2013). In contrast, overexpression of Sse1 or Ssb1 did not affect the rate of ΔssCPY^∗^-GFP degradation. The finding that Sse1 and Ssb1 do not affect UPS-mediated degradation of ΔssCPY^∗^-GFP, while preventing protein aggregation, further confirms that a defect in UPS-mediated degradation does not account for the high levels of protein aggregation in a *hac1* mutant. Together, these findings are in agreement with the idea that limitations in chaperone availability account for the increased protein aggregation in ER stress mutants.

### ER stress increases the spontaneous frequency of prion formation

**Figure 4.**
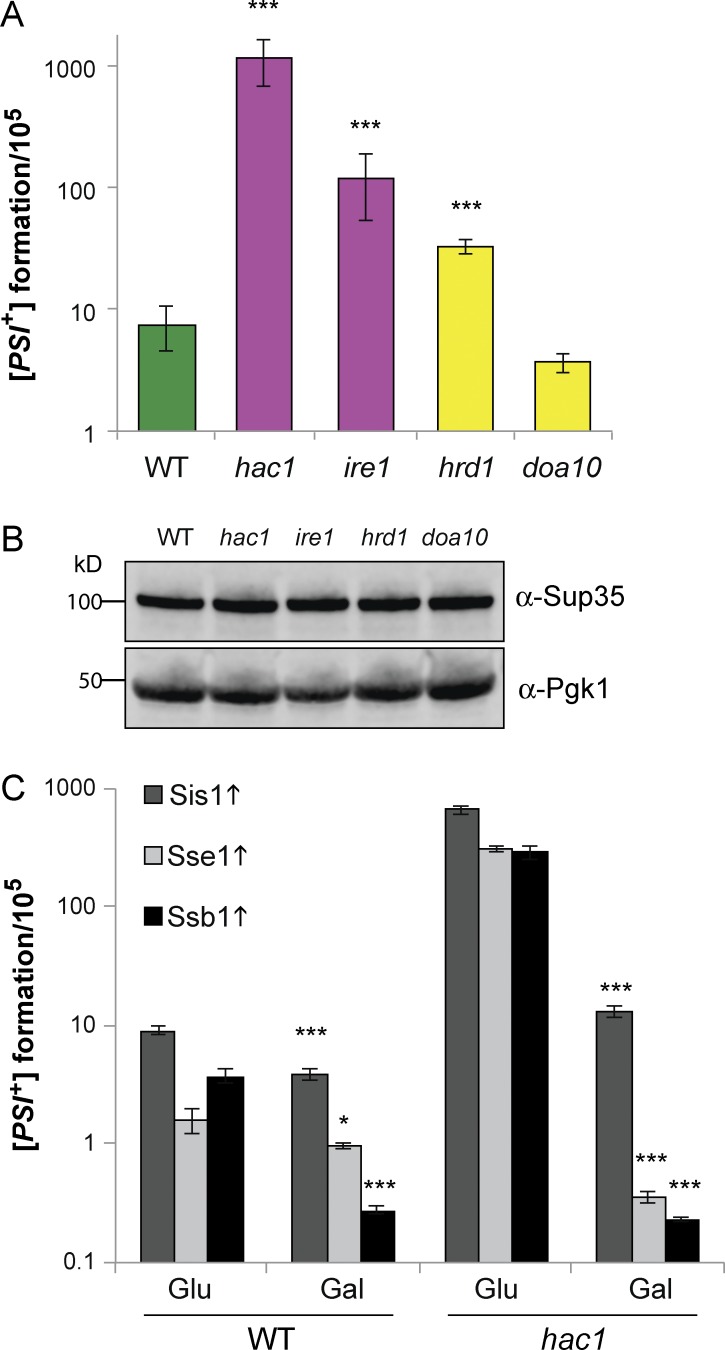
**The frequency of [*PSI*^+^] formation is increased in UPR and ERAD mutants.** (A) The frequency of [*PSI*^+^] prion formation was quantified in wild-type, *hac1, ire1, hrd1,* and *doa10* mutant strains. Data shown are the means of at least three independent biological repeat experiments ± SD. (B) Western blots probed with αSup35 or αPgk1. (C) The frequency of [*PSI*^+^] prion formation was quantified after the induction of the indicated chaperones (*n* = 3) ± SD. Significance is shown comparing strains grown on glucose media with strains grown on SGal media; *, P < 0.05; ***, P < 0.005 (*n* = 3).

A genome-wide screen for factors that increase [*PSI*^+^] prion induction identified mutants in the UPR and ERAD pathways (Tyedmers et al., 2008), suggesting that prion formation may also be a consequence of ER stress. We examined prion formation in our mutants and found that the frequency of [*PSI*^+^] prion formation was elevated in *hac1, ire1*, and *hrd1* mutants compared with wild-type and *doa10* mutant strains (Fig. 4 A). This increase in [*PSI*^+^] prion formation did not arise because of differences in Sup35 protein levels (Fig. 4 B). We examined whether the chaperones that abrogate protein aggregation in a *hac1* mutant could also prevent spontaneous prion formation and found that overexpression of Sis1, Sse1, and Ssb1 significantly reduced the frequency of [*PSI*^+^] prion formation in a *hac1* mutant (Fig. 4 C).

Overexpression of Sis1, Sse1, and Ssb1 reduced both amorphous and amyloid aggregation. Sis1 is an Hsp40 chaperone that regulates the ATPase activity of Hsp70 chaperones. It has been implicated in targeting misfolded proteins for degradation, facilitating the transfer of misfolded proteins to the nucleus, where they are degraded by the UPS system (Park et al., 2013; Shiber et al., 2013; Summers et al., 2013). Sis1 therefore appears to be an essential but limiting factor for degrading misfolded and aggregated proteins. Ssb1 is a ribosome-associated chaperone required to prevent the aggregation of nascent polypeptides (Koplin et al., 2010; Preissler and Deuerling, 2012). Ssb1 reduced aggregation in a *hac1* mutant, suggesting that proteins may misfold during translation, which would be particularly acute for highly abundant/translated proteins. Sse1 functions as a nucleotide exchange factor for Hsp70 chaperones (Easton et al., 2000; Shaner et al., 2005). Sse1 can bind unfolded peptides and suppress thermal aggregation by maintaining proteins in a folding-competent state (Oh et al., 1999). Sse1 is not thought to functionally refold proteins and hence may suppress protein aggregation in a *hac1* mutant via its holdase function.

### Chaperone overexpression rescues the sensitivity of a *hac1* mutant to ER stress

**Figure 5.**
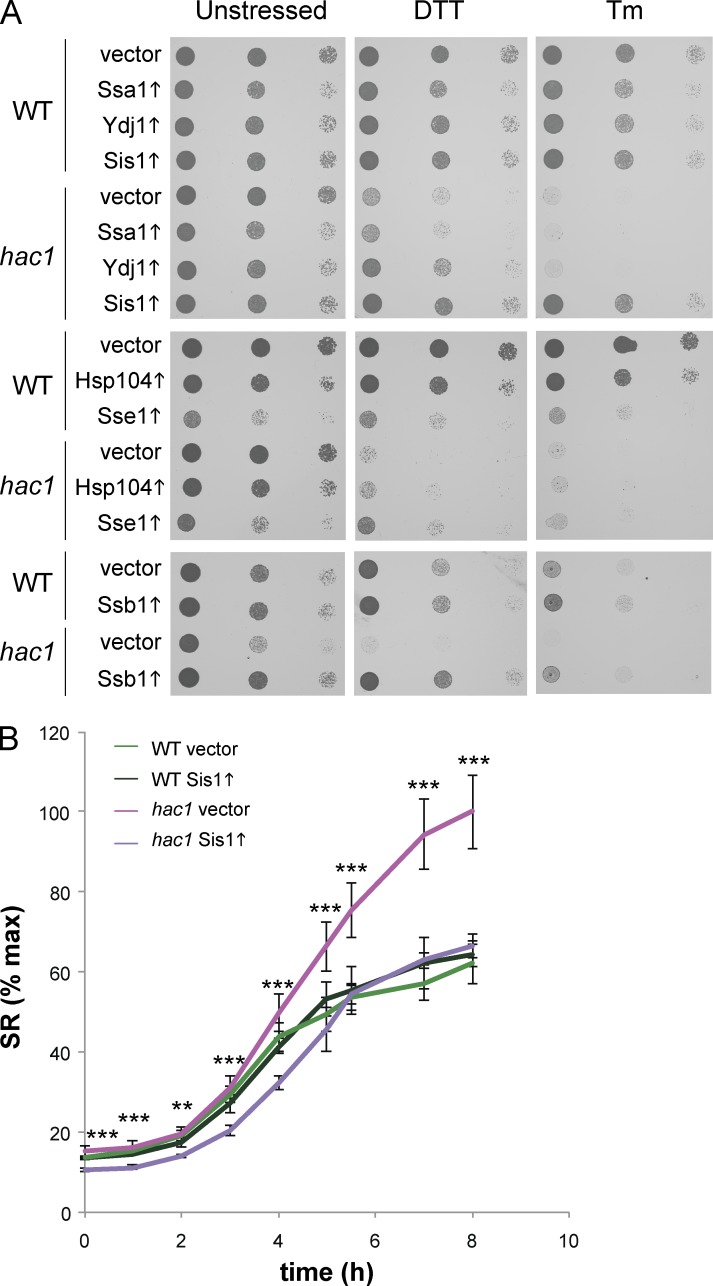
**Overexpression of selected chaperones rescues the sensitivity of a *hac1* mutant to ER stress.** (A) Strains as shown in Fig. 4 D were spotting onto SGal media containing DTT or Tm. (B) Wild-type and *hac1* mutant cells expressing GFP splicing reporter (SR) and containing a galactose-regulatable expression plasmid for Sis1 or empty vector (pRS413) were treated with 0.2 µg/ml Tm for 8 h. Fluorescence was measured by flow cytometry and is expressed relative to 100% maximum ± SD. Significance is shown comparing *hac1* vector with *hac1 SIS1* (*n* = 3). **, P < 0.01; ***, P < 0.005.

Because UPR mutants are sensitive to chemicals that promote ER stress, we tested whether the chaperones that abrogate protein aggregation influence the sensitivity of a *hac1* mutant to chemically induced ER stress (Fig. 5 A). Overexpression of Ssa1 or Hsp104 did not affect the sensitivity of the *hac1* mutant to DTT or Tm. Ydj1 minimally restored DTT tolerance but did not improve resistance to Tm stress. Sse1 was found to increase the sensitivity of the wild-type strain to DTT and Tm stress, whereas it increased the resistance of a *hac1* mutant to DTT stress and did not alter its sensitivity to Tm. The strongest effects were seen with Sis1 and Ssb1 overexpression, which rescued the sensitivity of a *hac1* mutant to both DTT and Tm; for Sis1, this resistance was comparable to wild-type levels (Fig. 5 A). We questioned whether chaperones rescue the *hac1* mutant by enabling it to cope with the same amount of ER stress or by diminishing the level of ER stress. We tested this using a splicing reporter (SR) where the *HAC1* open reading frame has been replaced with GFP (Pincus et al., 2010). This allows Ire1 activity to be monitored, because it splices the Hac1 intron to derepress GFP expression. We observed higher levels of SR activation in a *hac1* mutant than in the wild-type upon Tm stress (Fig. 5 B). Overexpression of Sis1 dampened the increased SR induction in the *hac1* mutant, indicating that Sis1 diminishes ER stress presumably by removing cytoplasmic protein aggregates.

In summary, our results demonstrate that ER stress causes defects in cytosolic protein homeostasis and the formation of protein aggregates. Protein aggregation occurs when proteins adopt aberrant conformations or misfold resulting in their abnormal association into larger, often insoluble structures (Hartl et al., 2011; Hipp et al., 2014). ER stress appears to lead to the aggregation of aggregation-prone proteins rather than the aggregation of ER or secretory pathway proteins. Most of the commonly aggregated proteins tended to be abundant, highly translated, stable proteins, in agreement with the idea that protein abundance is a good indicator of protein aggregation in vivo (Weids et al., 2016). It is generally thought that highly expressed and abundant proteins have evolved to be highly soluble and resistant to aggregation (Tartaglia et al., 2007; Castillo et al., 2011; Gsponer and Babu, 2012; Bednarska et al., 2013). However, there is relatively little flexibility in this equilibrium, and any conditions that alter protein solubility or concentration can promote aggregation (Tartaglia et al., 2007).

Mutants deleted for *HAC1* are sensitive to conditions that cause unfolded proteins to accumulate in the ER (Cox et al., 1993; Mori et al., 1993). Induction of gene expression by Hac1 promotes tolerance to ER stress by increasing ER chaperone concentrations. It is therefore surprising that overexpression of cytoplasmic chaperones rescued the sensitivity of a *hac1* mutant to conditions which cause unfolded protein accumulation in the ER. This suggests that in the absence of active UPR, ER stress causes cellular toxicity via cytoplasmic protein aggregation. There are established links between cytosolic chaperones and prevention of ERAD substrates from aggregation before degradation (Nishikawa et al., 2005). Hence, clearance or prevention of cytosolic aggregates may explain this stress rescue. In agreement, the Hac1 SR indicated that overexpression of Sis1 reduces ER stress caused by Tm exposure, and restored wild-type levels of ER stress tolerance to a UPR mutant. This new finding links the toxicity of ER stress with cytosolic protein aggregation and emphasizes the importance of interorganelle cross talk.

## Materials and methods

### Yeast strains and plasmids

The wild-type yeast strain 74D-694 (*MATa ade1-14 ura3-52 leu2-3,112 trp1-289 his3-200* [*PIN*^+^][*psi*^−^]) was used for all experiments. Strains were deleted for *HRD1* (*hrd1::HIS3, hrd1::TRP1*), *DOA10* (*doa10::HIS3*), *IRE1* (*ire1::LEU2*), and *HAC1* (*hac1::HIS3, hac1::TRP1*) using standard yeast methodology. Plasmids expressing fluorescently tagged proteins, including *Hsp104-RFP*, *Sis1-GFP*, and ΔssCPY^∗^-GFP (Lee et al., 2010; Malinovska et al., 2012; Park et al., 2013) and Hmg2-Myc (Hampton and Rine, 1994), have been described previously. Chaperones were overexpressed under the control of the *GAL1* promoter, including pESC-Leu-SSA1 (Park et al., 2013), pESC-Leu-YDJ1 (Park et al., 2013), pRS415GAL-HA-SIS1 (Park et al., 2013), p425GAL1-HSP104 (O’Driscoll et al., 2015), p425GAL1-SSE1 (O’Driscoll et al., 2015), and pAG416GAL-SSB1 (Malinovska et al., 2012). The GFP SR used to monitor Ire1 activity has been described previously (Pincus et al., 2010).

### Growth and stress conditions

Yeast strains were grown at 30°C with shaking at 180 rpm in rich YEPD medium (2% wt/vol glucose, 2% wt/vol bactopeptone, and 1% wt/vol yeast extract) or minimal SD (0.67% wt/vol yeast nitrogen base without amino acids and 2% wt/vol glucose) supplemented with appropriate amino acids and bases. SRaf media contained 2% wt/vol raffinose, and SGal media contained 1% wt/vol galactose/1% wt/vol raffinose in place of glucose. Media were solidified by the addition of 2% (wt/vol) agar. For chaperone expression, cultures were initially grown in SRaf media to exponential phase before switching to SGal media for a further 24 h to induce *GAL1* expression. For ER stress conditions, cells were treated with 2 mM DTT or 0.2 µg/ml Tm for 2 h. Stress sensitivity was determined by growing cells to stationery phase in SRaf media and spotting diluted cultures (A_600_ = 1.0, 0.1, 0.01) onto SGal agar plates containing various concentrations of DTT or Tm.

### Analysis of insoluble protein aggregates

Insoluble protein aggregates were isolated by separating insoluble proteins from soluble proteins by differential centrifugation and removing any contaminating membrane proteins using detergent washes (Weids and Grant, 2014). In brief, 20 ODs of cells were harvested by centrifugation at 4,000 rpm, 4°C, for 10 min. Cells were washed in 1 ml aggregate lysis buffer (ALB; 50 mM potassium phosphate, 1 mM EDTA, 5% (vol/vol) glycerol, 1 mM PMSF, and 1× complete mini-protease cocktail; Roche). Cells were resuspended in 300 µl ALB and spheroplasts were generated after treatment with 1 mg/ml lyticase for 30 min at 30°C. Cell breakage was achieved by sonication (Sonifier 150; Branson; 8 × 5 s, level 4), and samples were adjusted to equal protein concentrations before isolation of protein aggregates by centrifugation at 13,000 rpm, 4°C for 20 min. Insoluble fractions were resuspended in a buffer containing ALB buffer containing 2% (vol/vol) Igepal CA-630 (Sigma-Aldrich) through sonication (4 × 5 s, level 4). Samples were centrifuged at 13,000 rpm for 20 min at 4°C and the detergent wash repeated. Residual detergent was removed by two washes with ALB and the pellet resuspended by sonication. The final insoluble fraction was resuspended in 80 µl ALB and 20 µl reducing 4× protein loading buffer, separated by reducing SDS/PAGE (10% gels), and visualized by silver staining using the silver stain plus kit (Bio-Rad).

### MS and statistical analysis

Aggregated proteins were identified by MS (performed by the Biomolecular Analysis Core Facility, The University of Manchester) in triplicate for each condition. For protein identification, protein samples were run a short distance into SDS-PAGE gels and stained using colloidal Coomassie blue (Sigma-Aldrich). Total proteins were excised; trypsin was digested and identified using liquid chromatography MS. Proteins were identified using the Mascot mass fingerprinting program (http://www.matrixscience.com) to search the NCBInr and Swissprot databases. Final datasets for each condition were determined by selecting proteins that were identified in at least two of the three replicates. We identified 688, 820, and 818 proteins that aggregate in the wild-type, *hac1*, and *hrd1* mutants strains, respectively. Venn diagrams and analysis of the distribution of protein hits between different strains was performed using Venny (http://bioinfogp.cnb.csic.es/tools/venny/). Significantly enriched (5% false discovery rate) functional categories were identified using the MIPS Functional Catalogue (Ruepp et al., 2004). Datasets for each condition were assessed for functional enrichment (P < 0.01) of functional categories (MIPS database) using FunCat (available at http://www.helmholtz-muenchen.de/en/ibis). Mann–Whiney *U* tests were used to assess the statistical significance of observed differences in protein abundance (molecules per cell; Ghaemmaghami et al., 2003), estimated translation rates per protein (Arava et al., 2003), CAI, GRAVY score, pI, and protein stability (Christiano et al., 2014).

### Western blot analysis

The turnover of ΔssCPY^∗^-GFP and Hmg2-myc was assessed in cells by inhibiting protein synthesis with cycloheximide. Cells were collected at the indicated time points and ΔssCPY^∗^-GFP and Hmg2-myc levels analyzed by immunoblotting and quantified by densitometry. Values shown are percentages relative to zero time points from three independent biological repeats. Protein extracts were electrophoresed under reducing conditions on NuPAGE minigels (Thermo Fisher Scientific) and electroblotted onto polyvinylidene fluoride membrane (GE Healthcare). Primary antibodies used were rabbit α-Sup35 (Ness et al., 2002), ubiquitin (sc-8017; Santa Cruz Biotechnology, Inc.), Ydj1 (ab74442; Abcam), Sis1 (COP-080051; Operon Biotechnologies), Ssa1 (ADI-SPA-822; Enzo Life Sciences), GFP (A6465, Invitrogen), Pgk1 (459250; Thermo Fisher Scientific), Hsp104 (ab2924; Abcam), rabbit α-Sse1 (Chiabudini et al., 2012), and Myc 4A6 (05–724; EMD Millipore).

### Fluorescence microscopy

Sites of protein aggregation were detected using fluorescently tagged chaperones. Strains transformed with Hsp104-RFP or Sis1-GFP plasmids were harvested at the indicated time points and resuspended in water. The cells were immediately placed on poly-l-lysine–coated slides (Sigma-Aldrich) and visualized at room temperature using a IX71 (Olympus) Delta Vision (Applied Precision Ltd.) microscope with a 100×/NA.140 UPlan SAPO (oil) objective and FITC (BP490/20, BP531/28) and TRITC (BP555/28, BP617/63) band-pass filters from the Sedat QUAD filter set (Chroma Technology Corp.). Images were acquired using a Coolsnap HQ2 (Photometrics) camera using a Z optical spacing of 0.2 to 0.5 µm with Softworx 5.5.1 (Applied Precision Ltd.) and were then deconvolved with measured point spread function and maximum intensity quick projections were generated using the same software. For display, images were processed and analyzed using ImageJ.

### Analyses of prion formation

Yeast strain 74D-694 (*MATa ade1-14 trp1-289 his3-200 ura3-52 leu2-3,112*) contains an assayable nonsense (UGA) mutation in the *ADE1* gene. The induction of [*PSI*^+^] prion formation was quantified using the *ade1-14* mutant allele, which confers adenine auxotrophy and prions differentiated from nuclear gene mutations by their irreversible elimination in guanidine hydrochloride (GdnHCl). GdnHCl blocks the propagation of yeast prions by inhibiting the key ATPase activity of Hsp104, a molecular chaperone that is absolutely required for yeast prion propagation (Ferreira et al., 2001; Jung and Masison, 2001). The frequency of spontaneous [*PSI*^+^] prion formation was scored by growth in the absence of adenine. Diluted cell cultures were plated onto SD plates lacking adenine (SD-Ade) and incubated for 7–10 d. Colonies which grew on SD-Ade plates were counted and then picked onto new SD-Ade plates before replica-printing onto SD-Ade and SD-Ade containing 4 mM GdnHCl. Colonies that grew on SD-Ade, but not on SD-Ade with GdnHCl, were scored as [*PSI*^+^]. [*PSI*^+^] colonies were also scored by visual differentiation of red/white colony formation on YEPD plates and by the conversion of pink/white [*PSI*^+^] colonies to red [*psi*^−^] colonies on YEPD plates containing GdnHCl. Data shown are the means of at least three independent biological repeat experiments expressed as the number of colonies per 10^5^ viable cells.

### SR assay

Cells were grown in SGal media and treated with 0.2 μg/ml Tm for 8 h. Samples were collected hourly, and 10,000 cells were analyzed in an LSR Fortessa Flow cytometer (BD) with excitation at 488 nm and emission at 530/30 nm. The mean median value of the intensity of fluorescence of three independent biological repeats was calculated. The mean median value of the intensity of the *hac1* vector strain at the 8-h time point was considered the maximum and set to 100%. All other values were expressed relative to that.

### Statistical analyses

Data are presented as mean values ± SD. Statistical analysis for multiple groups was performed using one-way ANOVA with pairwise comparisons of sample means via the Turkey HSD test, and results were considered statistically significant with a p-value <0.05. The physicochemical, translation, and degradation rates of proteins in aggregates were evaluated with pairwise Mann–Whitney–Wilcoxon *U* tests.

### Online supplemental material

Fig. S1 presents MIPS functional categorization of aggregated proteins identified in the wild type and *hac1* and *hrd1* mutant strains. Fig. S2 presents MIPS functional categorization of aggregated proteins identified in the common, *hac1*-only, and *hrd1*-only sets. Fig. S3 A presents the proportion of proteins in each aggregate set that interact with specific chaperones, and Fig. S3 B presents immunoblot analysis to confirm overexpression of selected chaperones.

## Supplementary Material

Supplemental Materials (PDF)
