# Supplementary material for: ER stress causes widespread protein aggregation and prion formation
Source: J Cell Biol. 2017 Aug 7;216(8):2295–304. doi: 10.1083/jcb.201612165 (PMC5551711; doi:10.1083/jcb.201612165)
Supplement: Supplemental Materials (PDF) [file JCB_201612165_sm.pdf]

Hamdan et al., https://doi.org/10.1083/jcb.201612165

THE JOURNAL OF CELL BIOLOGY

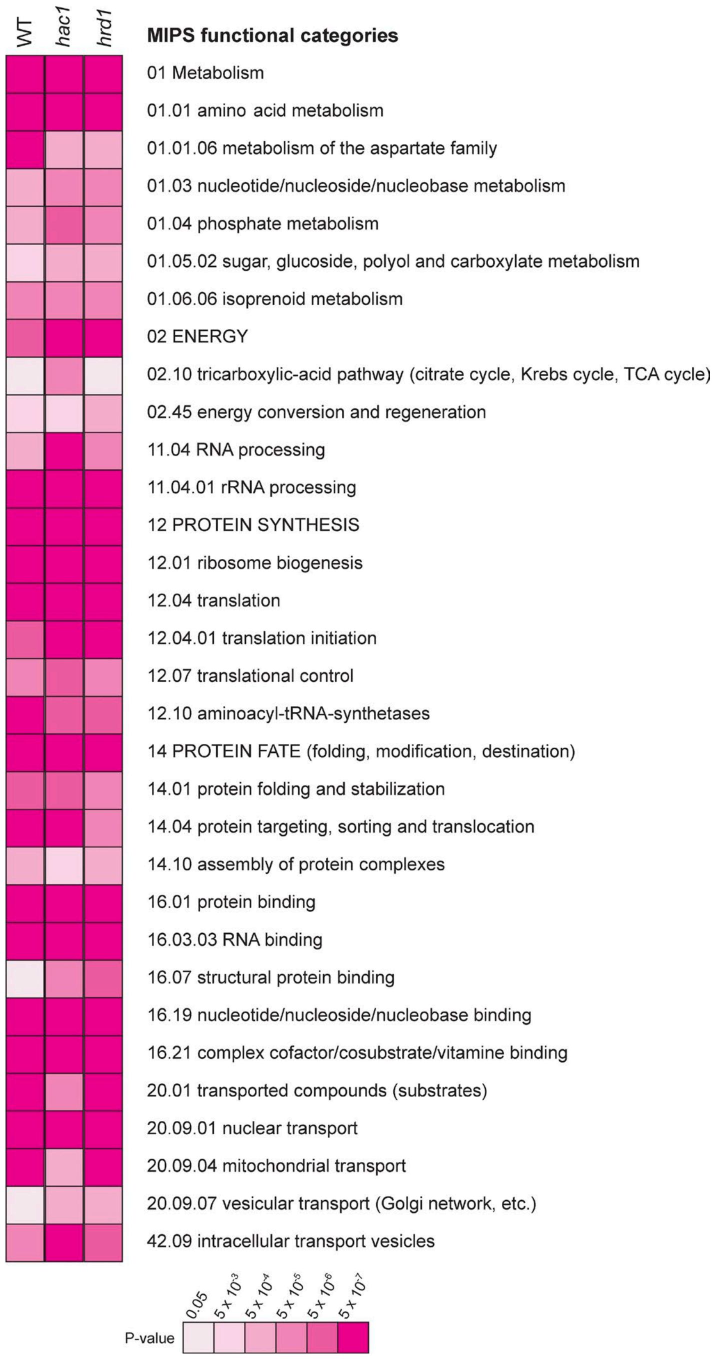

Figure S1. **MIPS functional categorization of aggregated proteins identified in the wild-type, *hac1*, and *hrd1* mutant strains.** Significantly enriched functional categories within the data sets were determined using FunCat (false discovery rate < 5%). Results are ordered on MIPS category classification numbers, and overarching categories are in capitals. Confidence of each classification category is shown as Bonferroni-corrected p-values.

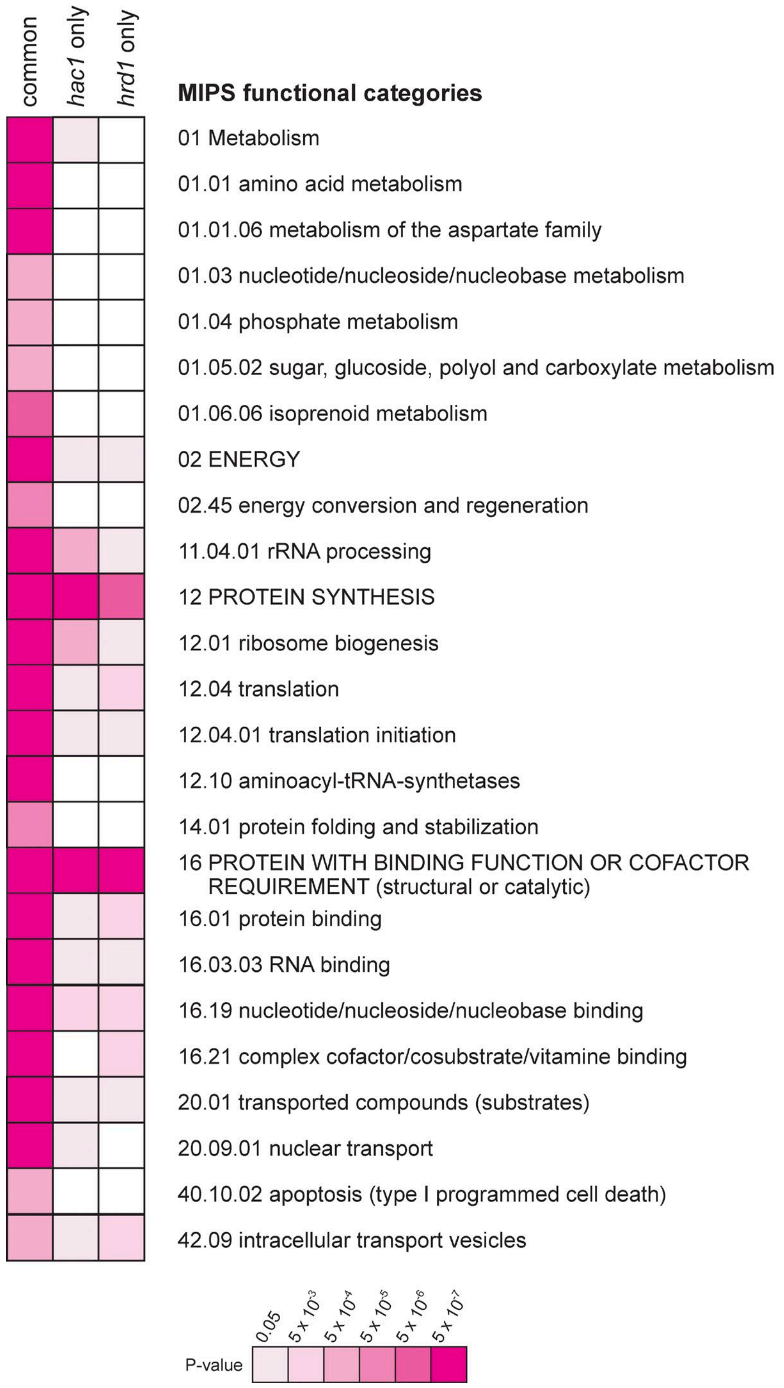

Figure S2. **MIPS functional categorization of aggregated proteins identified in the common, *hac1*-only, and *hrd1*-only sets.** Significantly enriched functional categories within the data sets were determined using FunCat (false discovery rate <5%). Results are ordered on MIPS category classification numbers, and overarching categories are in capital letters. Confidence of each classification category is shown as Bonferroni-corrected p-values.

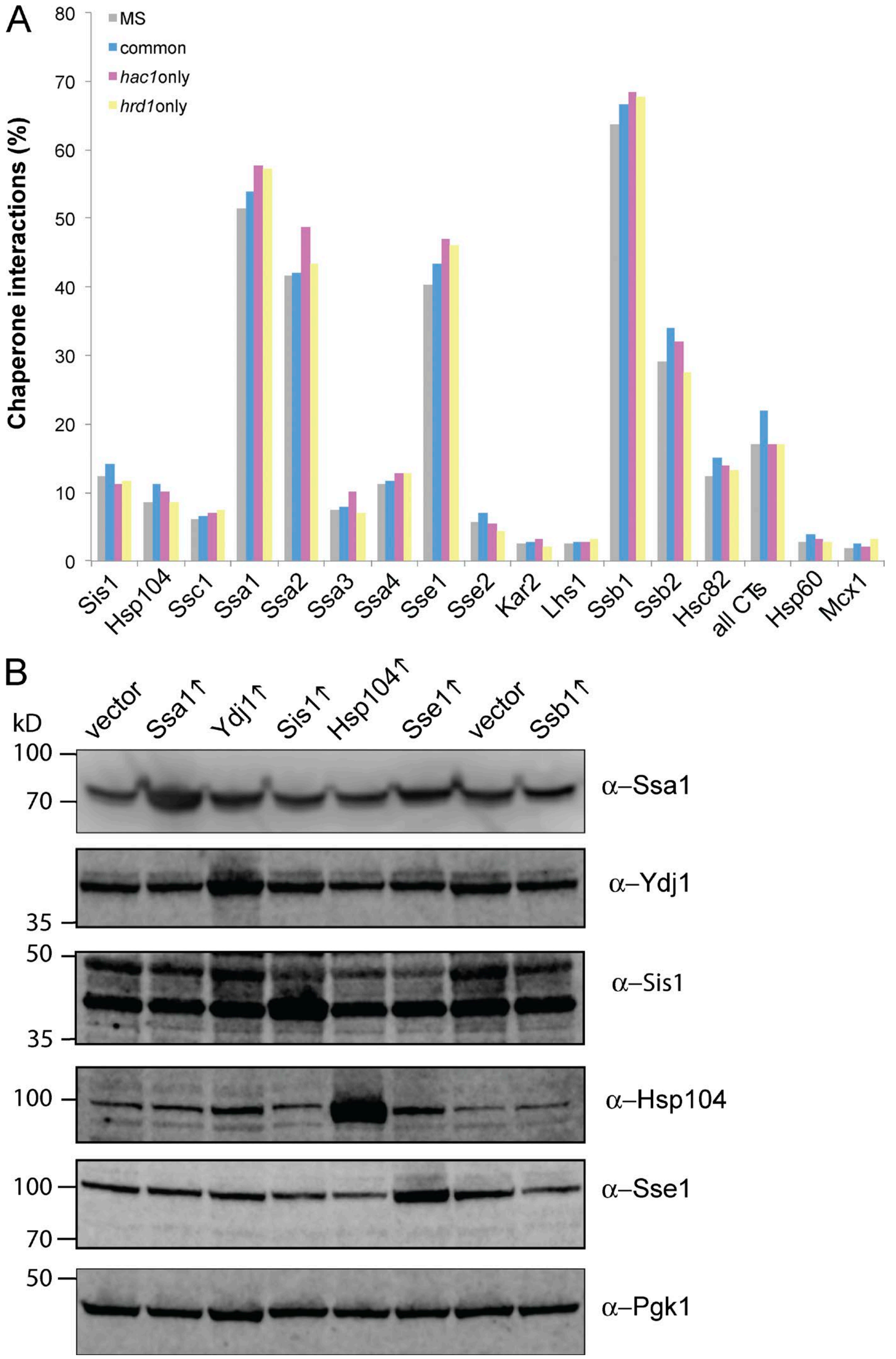

Figure S3. **Analysis of chaperone interactions and concentrations in UPR mutants.** (A) The proportion of proteins in each aggregate set that interact with specific chaperones is shown. Chaperone–protein interaction data were retrieved from Gong et al. (2009), and the number of chaperones interactions per protein was scored. The distributions for each dataset was calculated, and no enrichment is found for proteins with extensive chaperone interactions in the aggregated proteins (common set, *hrd1*-only, and *hac1*-only) compared with unaggregated proteins in the MS proteome. (B) Western blot analysis of the *hac1* mutant containing galactose-regulatable expression plasmids for Ssa1, Ydj1, Sis1, Hsp104, Sse1, or Ssb1. Vector denotes an empty vector control, which was pRS413 (Ssa1, Ydj1, Sis1, Hsp104, and Sse1) or pRS415 (Ssb1). Cultures were initially grown in SRaf media to exponential phase before switching to SGal media for a further 24 h to induce GAL1 expression. Blots were probed with specific antibodies as indicated. We could not examine Ssb1 because of the lack of an available antibody.

Reference

Gong, Y., Y. Kakihara, N. Krogan, J. Greenblatt, A. Emili, Z. Zhang, and W.A. Houry. 2009. An atlas of chaperone-protein interactions in *Saccharomyces cerevisiae*: implications to protein folding pathways in the cell. *Mol. Syst. Biol.* 5:275. http://dx.doi.org/10.1038/msb.2009.26
